# Reaction of Commercial Cultivars of Kiwifruit to Infection by Root-knot Nematode and Its Biocontrol Using Endophytic Bacteria

**DOI:** 10.2478/jofnem-2023-0020

**Published:** 2023-06-05

**Authors:** Seyedeh Najmeh Banihashemian, Salar Jamali, Morteza Golmohammadi, Sina Noorizadeh, Mohammad Reza Atighi

**Affiliations:** Plant Protection Department, Faculty of Agricultural Sciences, University of Guilan, Rasht, Iran; Horticultural Science Research Institute, Citrus and Subtropical Fruits Research Center, Agricultural Research Education and Extension Organization (AREEO), Ramsar, Iran; Plant Protection Department, Agriculture Faculty, Tabriz University, Tabriz, Iran; Department of Plant Pathology, Faculty of Agriculture, Tarbiat Modares University, Tehran, Iran

**Keywords:** cultivars, endophytic bacteria, kiwifruit, root-knot nematode

## Abstract

Root-knot nematodes (RKN) cause considerable economic losses to kiwifruit production annually. Screening of resistant cultivars has been one of the long-standing methods to manage root-knot nematodes. Here, the reaction of the four most common commercial cultivars of kiwifruit, namely, *Actinidia chinensis* var. *deliciosa* cv. Hayward, *A. chinensis* var. *deliciosa* cv. Abbott, *A. chinensis* var. *deliciosa* cv. Bruno, and *A. chinensis* var. *chinensis* cv. Haegeum (commonly known as ‘Golden’ kiwifruit) to infection by the RKN, *Meloidogyne incognita*, was evaluated. Among examined cultivars ‘Golden’ was the most susceptible, having on average 52.8 galls, 56.1 egg masses per gram of root, and 642 J2 population per 200 gram of soil. ‘Bruno’ showed the highest resistance, with 3.3 galls, 4.1 egg masses per gram of root, and 79 J2 in 200 g of soil. Then, two potential biological control agents, namely *Priestia megaterium* 31.en and *Agrobacterium tumefaciens* 19.en were used on ‘Hayward’ seedlings against *M. incognita* and showed a significant reduction in the number of galls and egg masses on roots, juvenile population in the soil, and increased the growth parameters of the plants compared to non-treated seedlings. We demonstrated that integrated management using resistant cultivars and biological control can provide a safe and economic method to control RKN, and these resistant cultivars can be used in breeding programs.

Kiwifruit (*Actinidia* spp. ‘Lindl’) is one of the most important horticultural crops around the world. It is important not only for its nutritional properties, e.g., high in antioxidants and vitamin C content, but also for its application for the treatment of cancer, hepatitis, and cardiovascular disease (Li et al., 2016; Pan et al., 2020). The genus *Actinidia* includes more than 70 species (Garcia et al., 2011; Ma et al., 2021) and is distributed around the world, especially in China, Italy, New Zealand, Chile, Greece, and Iran (Ferguson, 2016). The Food and Agriculture Organization (FAO) reported that the production of kiwifruit in the world was about 4.4 million tons in 2021. Iran, with a production of 294,000 tons, was ranked fifth (FAOSTAT, 2021). Among the major commercial kiwifruits varieties like *A. chinensis* var. *deliciosa* cv. Hayward (green kiwifruit) and *A. chinensis* var. *chinensis* cv. Haegeum (‘Golden’ kiwifruit), Hayward is the most common commercial cultivar in Iran (Maghdouri et al., 2021).

Soil-borne pathogens cause a significant yield loss on kiwifruit annually and among them, root-knot nematodes (RKN), *Meloidogyne* spp., are one of the most important pathogens. Four species of RKN, including *M. incognita*, *M. javanica*, *M. arenaria*, and *M. hapla* have been reported from Iran and *M. incognita* (*Mi*) is known as the major species infecting kiwifruit orchards of Mazandaran Province, the biggest kiwifruit production region in Iran (Tanha Maafi and Mahdavian, 1997; Banihashemian et al., 2022). Management of nematodes is difficult because of their wide host range and survival in diverse environmental conditions. Using chemical nematicides is the most common management method for nematodes. Due to the extensive use of pesticides and their deleterious effects on the environment and human health, their use has been banned or limited. Hence, using eco-friendly methods like resistant cultivars and biological control have attracted special attention recently (Vetrivelkalai, 2019; Eliwa and Hagag, 2021). The use of nematode-resistant cultivars along with biological control in integrated management programs provides a suitable strategy to control RKN (Mukhtar et al., 2014; Mukhtar, 2018; Eliwa and Hagag, 2021). Resistant cultivars have been successfully used against RKN in tomato (Lizardo et al., 2022), soybean (Izuogu et al., 2015), pepper (Bello et al., 2015) and even provided the complete-spectrum resistance in the case of Myrobalan plum (Claverie et al., 2011). Using biological control agents against plant pathogens, especially plant parasitic nematodes where resistant cultivars are not available or in combination with partially resistant cultivars and other control strategies provides effective and durable control against nematodes.

Endophytic bacteria employ a diversity of mechanisms to promote plant growth or protect plants against pathogens. Directly, they provide more nutrients that are required for plants, especially in soils with poor content of nitrogen, iron, and phosphorus. Production of phytohormones like auxin, gibberellic acid, ethylene, cytokinin, and abscisic acid not only promote plant growth but also are involved in plant defense against pathogens (Gamalero and Glick, 2011; Rosier et al., 2018; Ali et al., 2021). They may indirectly protect and promote plant growth by nutrient competition, antibiotic production, and induction of resistance against pathogens (Ali et al., 2021). The *Bacillus cereus* strain D13 protects rice plants against *Xanthomonas oryzae* pv. *oryzae* by producing a range of volatile compounds like 3,5,5-trimethylhexanol and decyl alcohol (Xie et al., 2018). It has been shown that *Priestia megaterium* strain JR48 induces resistance against *Xanthomonas campestris* pv. *campestris* in cruciferous plants by induction of hydrogen peroxide accumulation, callose deposition, and elevated expression of defense-related genes, especially pathogenesis-related (*PR*) genes through the salicylic acid signaling pathway (Li et al., 2022). Application of *Streptomyces* sp. on banana provided a biocontrol efficiency of 70.7% against *Meloidogyne javanica* (Su et al., 2017). It has been shown that treatment of kiwifruit seedlings with *Pantoea ananatis* and *Pseudomonas chlororaphis* causes a significant reduction in the number of galls and egg masses. Moreover, they significantly increased growth parameters, including root-fresh and shoot-fresh weight compared to non-treated kiwifruit seedlings (Banihashemian et al., 2022).

Here, we first investigated the resistance of four common commercial cultivars of kiwifruit, including *A. chinensis* var. *deliciosa* cv. Hayward, *A. chinensis* var. *deliciosa* cv. Abbott, *A. chinensis* var. *deliciosa* cv. Bruno, and *A. chinensis* var. *chinensis* cv. Haegeum (commonly known as ‘Golden’ kiwifruit) to *Mi.* Also, the antagonistic potential of two endophytic bacteria namely *Priestia megaterium* 31.en and *Agrobacterium tumefaciens* 19.en against *Mi* was evaluated.

## Materials and Methods

### Preparation of nematode inoculum

The *Mi* population, which was previously isolated from infected roots of kiwifruit plants in the Mazandaran provinces, Iran, was used in this study (Banihashemian et al., 2022). Briefly, a single egg mass, isolated from infected kiwifruit roots, was propagated and maintained on roots of susceptible tomato plants (Early Urbana variety) under greenhouse conditions (temperature of 25°C ± 2 and RH of 70%). After three months, egg masses were hand-picked under a stereomicroscope (Nikon, SMZ800) and were left to hatch at 25°C for five to seven days using small trays according to the tray method (Whitehead and Hemming, 1965). The freshly hatched second-stage juveniles were then used for inoculation of plants.

### Plant material

The four most common commercial cultivars of kiwifruit cultivated in the north of Iran, namely, *A. chinensis* var. *deliciosa* cv. Hayward, *A. chinensis* var. *deliciosa* cv. Abbott, *A. chinensis* var. *deliciosa* cv. Bruno, and *A. chinensis* var. *chinensis* cv. Haegeum (‘Golden’ kiwifruit) were selected to evaluate their resistance to *Mi*. Seeds of kiwifruit plants were grown in an equal volume of the sterilized mixture of perlite, sand, and cocopeat in pots containing 1 k of soil under controlled temperature conditions (25°C±2) and 70% relative humidity (RH) in the greenhouse at the Citrus and Subtropical Fruits Center, Agricultural Research Education and Extension Organization (AREEO), Ramsar, Iran.

### Infection assay and measurement of plant growth parameters

The six-month-old seedlings of kiwifruit were inoculated with 2,000 second-stage juveniles of *Mi* through four holes in the soil around the roots of the seedlings. The control seedlings were inoculated with the same amount of water used for nematode inoculation.

Plants were watered every 7 days, and 50 days after inoculation, seedlings were uprooted, and the roots were washed gently under running water to clean off soil debris. The clean roots were stained by boiling in 0.01% acid fuchsin for 15 min, then destained in acid glycerol (100 ul of HCL in 100 ml of glycerol) for three weeks with a gentle constant shaking (80 rpm) (Atighi et al., 2021). The numbers of galls and egg masses per gram of root were counted under a stereomicroscope (Nikon SMZ800). Counting was repeated three times for each root system to make an average. The nematode gall index of kiwifruit seedlings was rated using a 0–5 scale according to Taylor and Sasser (1978). Some modifications in the rating scale based on the number of galls were used to evaluate the degree of resistance/susceptibility of cultivars (Table 1). Also, to count the number of J2s in the soil, all the soil in the pot was mixed thoroughly, and then 200 g of soil was used to extract nematode using the tray method (Whitehead and Hemming, 1965). In addition, the kiwifruit growth parameters, such as fresh and dry weight of root and shoot, were measured while plants were harvested. These experiments were conducted in a randomized block design with five biological replicates per experiment and repeated three times independently.

**Table 1.** Modified rating scale for the evaluation of the level of resistance/susceptibility of kiwifruit cultivars based on the number of galls according to (Taylor and Sasser, 1978).

**Scale**	**Number of Galls per Plant**	**Resistance Rating**
0	0	Immune (I)
1	1–2	Resistant (R)
2	3–10	Moderately Resistant (MR)
3	11–30	Moderately Susceptible (MS)
4	31–100	Susceptible (S)
5	>100	Highly Susceptible (HS)

### Preparation and characterization of bacterial antagonists

The endophytic isolates of *Priestia megaterium* strain 31.en (OK560186) and *Agrobacterium tumefaciens* strain 19.en (OK398382) had previously been isolated from seemingly healthy kiwifruit plants in orchards of Mazandaran and Guilan provinces, Iran (Banihashemian et al., 2022). Isolation of these two isolates was done according to the method of Wicaksono et al. (2018). The samples were sterilized with 96% ethanol for 10 seconds and 2% sodium hypochlorite solution for 3 min followed by washing three times (1 min each time) with sterile-distilled water in a laminar flow cabinet. The disinfected tissues were chopped into small pieces in sterilized water and kept for 30 to 40 min; then 30 μL of suspension was cultured on sucrose nutrient agar (NAS) medium after serial dilution. Also, 100 μl of the last wash was transferred to Luria-Bertani (LB; Tryptone 10 gr, NaCl 10 g, yeast extract 5 g, distilled water 950 ml) as a control (Taechowisan et al., 2003). The plates were incubated at 25°C for 7 days. The colonies were recultured on NAS plates until pure colonies were obtained. Single colonies were stored in 60% glycerol stock at −80°C for future studies.

### Inoculation of endophytic bacteria, *P. megaterium* strain 31.en and *A. tumefaciens* strain 19.en on plants and evaluation of plant growth parameters

The cultivar Hayward is the main cultivar grown in the north of Iran. The endophytic bacteria were cultured in liquid Luria-Bertani (LB) medium at 25°C with constant shaking at 200 rpm for 48 h to obtain a bacterial suspension. The culture of bacteria was centrifuged at 6,000 rpm for 5 min and washed three times with sterile deionized water. The achieved precipitates resuspended in sterilized water to an optical density (OD) value of 0.5 at 600 nm. To evaluate the efficacy of endophytic bacteria against *Mi*, six-month-old seedlings were inoculated with 40 ml suspension of the endophytic bacteria *P. megaterium* strain 31.en and *A. tumefaciens* strain 19.en (10^7^ CFU/ml) and after 2 days, 2,000 J2 of *Mi* were inoculated on roots of kiwifruit seedlings and kept at greenhouse conditions, as described above. The treatments were divided into four groups as follows: (i) treated with sterile water as control; (ii) inoculated only with the *Mi*; (iii) pretreated with *P. megaterium* strain 31.en and then inoculated with the *Mi*; and (iv) pre-treated with *A. tumefaciens* strain 19.en and then inoculated with the *Mi*.

After 50 days, the number of galls and egg masses, fresh and dry weights of shoot and root, and J2 population of nematodes in the soil were measured. The experiment was carried out in a randomized block design with five replications and repeated three times independently.

### Statistical analysis

Data were analyzed using SAS version 9.1 software with a one-way variance analysis (ANOVA) test. The mean ± standard deviation (X±SD) was calculated in all experimental data. The Bartlett's test was performed to check the equality of variances, so data were pooled to analyze them together. The significance of differences (*P* < 0.05) within treatments was determined using the least significant difference (LSD) test. For all treatments, there were five replicates, and the experiment was repeated three times independently.

## Results

### Response of kiwifruit cultivars to infection by *Mi*

**Figure 1:**
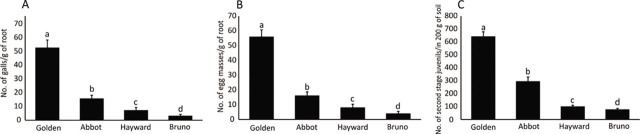
Reaction of commercial cultivars of kiwifruit to infection by *M. incogntia*. (A) Number of galls on roots; (B) Number of egg masses on roots; (C) Number of J2s in the soil. Different letters denote significant differences (*p* < 0.05). Error bars indicate STD. (n=15).

The response of the selected kiwifruit cultivars to infection by *Mi* is shown in Figures 1A–C. The greatest number of galls (52.8) and egg masses (56.1) per root system were observed in ‘Golden’ kiwifruit, while the minimum number of galls and egg masses were observed in Bruno and Hayward cultivars, respectively (Figs. 1A,B). The rating scale showed that the cultivar ‘Golden’ was susceptible to *M. incognita* with 31 to 100 galls per plant. Cultivar Abbot was moderately susceptible with 11 to 30 galls per plant. Bruno and Hayward were moderately resistant, with 3 to 10 galls per plant, and Bruno showed a significant resistance when compared to Hayward (Table 1 and Fig. 1A). Also, the number of J2 per 200 g of soil was significantly increased in ‘Golden’ kiwifruit inoculated with *Mi*, and the minimum number of J2 was seen in the Bruno cultivar (Fig. 1C).

**Figure 2:**
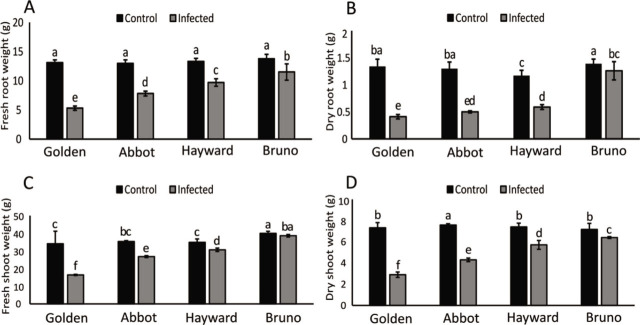
Growth parameters of plants in response to infection by *M. incogntia*. (A) Fresh root weight; (B) dry root weight; (C) fresh shoot weight; (D) dry shoot weight. Different letters denote significant differences (*p* < 0.05). Error bars indicate STD (n=15).

**Figure 3:**
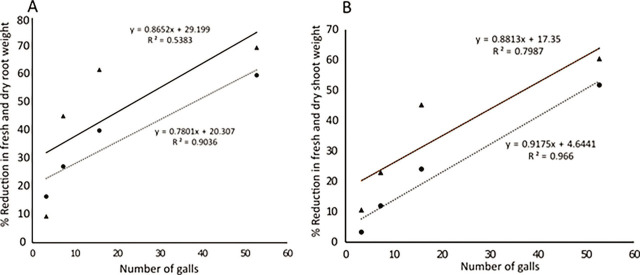
Relationships between the number of galls and percentage of reductions in fresh and dry root and shoot weights. (A) (●) and (▲) represent reductions in fresh and dry root weights of kiwifruit plants, respectively. (…) and (―) represent trend lines showing reductions in fresh and dry root weights of kiwifruit plants, respectively; (B): (●) and (▲) represent reductions in fresh and dry shoot weights of kiwifruit plants, respectively. (…) and (―) represent trend lines showing reductions in fresh and dry shoot weights of kiwifruit plants, respectively.

Likewise, fresh and dry root and shoot weights in all the cultivars were different from each other. The maximum reduction in fresh root and shoot weight was observed in the ‘Golden’ cultivar, and the maximum fresh and dry root and shoot weight was seen in the Bruno and Hayward cultivars, respectively (Figs. 2A–D). Also, regression analysis displayed positive relationships between the number of galls and reductions in dry and fresh root and shoot weights (Figs. 3A,B).

### Antagonistic effects of the endophytic bacteria *P. megaterium* strain 31.en and *A. tumefaciens* strain 19.en against *Mi* in greenhouse studies

**Figure 4:**
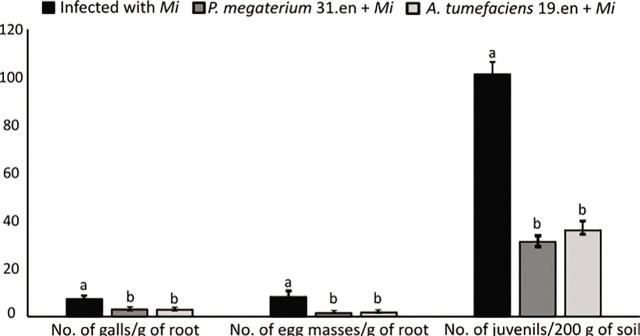
Antagonistic effects of *P. megaterium* strain 31.en and *A. tumefaciens* strain 19.en against *M. incognita* in kiwifruit under greenhouse conditions. Different letters denote significant differences (*p* < 0.05). Data are the mean of five replications which were repeated three times independently (n=15).

**Figure 5:**
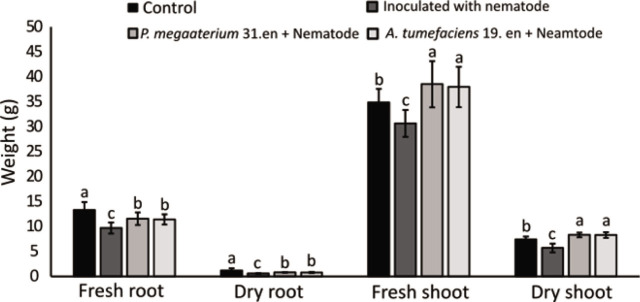
Effects of *P. megaterium* strain 31.en and *A. tumefaciens* strain 19.en on growth parameters of kiwifruit under greenhouse conditions. Different letters denote significant differences (*p* < 0.05). Data are the mean of five replications that were repeated three times independently (n=15).

The number of *Mi* galls and egg masses in the root system decreased in kiwifruit pretreated with *P. megaterium* strain 31.en and *A. tumefaciens* strain 19.en strains compared to the control plants (Fig. 4). Moreover, kiwifruit growth parameters including the fresh and dry weight of the shoot were increased (Fig. 5).

## Discussion

Infection of kiwifruits by *Mi* causes a significant reduction in its growth and production. Different measures are used to control nematodes and due to their difficult management, using integrated management methods, especially eco-friendly measures like the use of resistant cultivars and biocontrol agents, have attracted more attention recently and can provide benefits for both farmers and the environment. Using resistance cultivars against plant parasitic nematodes has been one of the long-lasting approaches to fend off their attack. To reach this end, screening resistance plants is the main step not only to be used in the infested areas and areas with high potential for infection by RKN, but also for breeding programs or as a rootstock for a highly productive scion (Saucet et al., 2016; Lesmes-Vesga et al., 2022; Mahoonaki et al., 2023). Also, the use of biological agents to control plant diseases has been the subject of much research due to the deleterious effect of chemical pesticides on human health and the environment. The integration of eco-friendly management approaches like resistant cultivars and biological agents provides an efficient management strategy to control diseases, especially for nematodes, due to difficulties in their management. Here, we provide what we believe is the first study evaluating the reaction of the four most common commercial cultivars of kiwifruit in Iran. Also, the biological control efficacy of two endophytic bacteria, namely, *P. megaterium* strain 31.en and *A. tumefaciens* strain 19.en against *Mi* was evaluated. Two out of four selected cultivars showed moderate resistance against infection by *Mi* according to scales provided by Taylor and Sasser (1978). The same result was observed previously where the resistance of three kiwifruit genotypes against several species of RKNs was evaluated, demonstrating that their genetic background influences their resistance against *Mi*. Notably, variation has been observed in the reproduction of *Mi* on kiwifruit cultivars in agreement with the previous study by Nicotra et al. (2003). This provides a new genetic resistant source against *Mi* that can be used in breeding programs or as rootstock for grafting highly productive scions in infested soils. It has been shown that in case of myrobalan plum, the woody plant host, the presence of the *Ma* gene, confers complete-spectrum resistance against *Meloidogyne* spp. by hypersensitive response during migration of second-stage juveniles in host tissue (Claverie et al., 2011; Saucet et al., 2016). The myrobalan plum has been used widely as both rootstock for grafting susceptible but highly productive plum trees or in breeding programs for the generation of new resistant plants (Salesses et al., 1998; Rubio-Cabetas et al., 1998; Lecouls et al., 2004). The reduction of growth parameters of the four kiwifruit cultivars compared to non-inoculated kiwifruits, especially in the susceptible cultivar, ‘Golden’, was consistent with a previous study on okra infected with *Mi* (Mukhtar et al., 2014). Here, the combination of both methods for cultivar Hayward resulted in a significant reduction of the severity of the disease as well as improved plant growth by increasing nutrient absorption and production of secondary metabolites (Moyes et al., 2016; Chen et al., 2017; Naylor et al., 2017; Liu et al., 2019).

Many endophytic bacteria have been reported to inhibit pathogens and promote the growth and health of plants (Maheshwari and Annapurna, 2017; Su et al., 2017; Hu et al., 2018; Santos et al., 2018; Tran et al., 2019; Vetrivelkalai, 2019). It is shown that *Priestia megaterium* (previously known as *Bacillus megaterium*) is used as a biocontrol agent against RKN (Elshafie et al., 2012; Mohammadi et al., 2017). *B. megaterium* DS9 reduced the nematode population in the soil and increased plant growth parameters in pepper (Tran et al., 2019). Also, *B. megaterium* had nematicidal activity against *Mi* and increased the growth parameters in sugar beet (Youssef et al., 2017). Moreover, *B. megaterium* increased the accessibility of available phosphorus in the soil to uptake by plants, enhanced the synthesis of organic matter in soil, and increased the growth parameters. They also caused antibiosis potential against the nematode activity (Mostafa et al., 2018). In agreement with our results, the application of endophytic *A. tumefaciens* strains as plant-growth-promoting bacteria was observed in other studies where endophytic *A. tumefaciens* CCNWGS0286 promoted the growth of *Robinia pseudoacacia* L. significantly (Hao et al., 2012). *A. tumefaciens* CR22 showed an antagonistic ability against *F. oxysporum* (Hernández-Pacheco et al., 2021). The endophyte *A. tumefaciens* showed the potential to control soybean diseases (de Almeida Lopes et al., 2018).

Here, we showed the response of kiwifruit cultivars to infection by *Mi.* The cultivars Bruno and Hayward were found to be moderately resistant, and Bruno showed a significant resistance when compared to Hayward. Moreover, we showed the antagonistic potential of two endophytic bacteria, *P. megaterium* strain 31.en and *A. tumefaciens* strain 19.en, against *Mi.* The integration of both resistant cultivars and biological control agents can provide a new eco-friendly strategy to manage RKN in kiwifruit orchards and reduces the negative effects of chemical pesticides on the environment and human health. Thus, this information is valuable for kiwifruit growers to use resistant plants in infested areas, especially in combination with endophytic bacteria. In our study, the number of galls, egg masses, and nematodes in Bruno and Hayward cultivars indicate the presence of moderate resistance, and its mechanisms remain to be elucidated. Also, which mechanisms by two endophytic bacteria are employed to decrease the negative effect on *Mi* infection needs to be explored.

## References

[j_jofnem-2023-0020_ref_001] Ali M., Ali Q., Sohail M. A., Ashraf M. F., Saleem M. H., Hussain S., Zhou L. (2021). Diversity and taxonomic distribution of endophytic bacterial community in the rice plant and its prospective. International Journal of Molecular Sciences.

[j_jofnem-2023-0020_ref_002] Atighi M. R., Verstraeten B., De Meyer T., Kyndt T. (2021). Genome-wide shifts in histone modifications at early stage of rice infection with *Meloidogyne graminicola*. Molecular Plant Pathology.

[j_jofnem-2023-0020_ref_003] Banihashemian S. N., Jamali S., Golmohammadi M., Ghasemnezhad M. (2022). Isolation and identification of endophytic bacteria associated with kiwifruit and their biocontrol potential against *Meloidogyne incognita*. Egyptian Journal of Biological Pest Control.

[j_jofnem-2023-0020_ref_004] Bello T., Fawole B., Claudius-Cole A. (2015). Susceptibility of seven varieties of pepper and tomato to root-knot nematodes (*Meloidogyne* spp.) in Ibadan. Journal of Agriculture and Veterinary Science.

[j_jofnem-2023-0020_ref_005] Chen S., Zhang M., Wang J., Lv D., Ma Y., Zhou B., Wang B. (2017). Biocontrol effects of *Brevibacillus laterosporus* AMCC100017 on potato common scab and its impact on rhizosphere bacterial communities. Biological Control.

[j_jofnem-2023-0020_ref_006] Claverie M., Dirlewanger E., Bosselut N., Van Ghelder C., Voisin R., Kleinhentz M., Lafargue B., Abad P., Rosso M.-N., Chalhoub B. (2011). The Ma gene for complete-spectrum resistance to *Meloidogyne* species in Prunus is a TNL with a huge repeated C-terminal post-LRR region. Plant Physiology.

[j_jofnem-2023-0020_ref_007] de Almeida Lopes K. B., Carpentieri-Pipolo V., Fira D., Balatti P. A., López S. M. Y., Oro T. H., Stefani Pagliosa E., Degrassi G. (2018). Screening of bacterial endophytes as potential biocontrol agents against soybean diseases. Journal of Applied Microbiology.

[j_jofnem-2023-0020_ref_008] Eliwa G. I., Hagag E. S. (2021). Approach to new peach rootstocks resistant to root-knot nematodes (*Meloidogyne* species) selected from local Mit-Ghamer peach cultivar. Scientia Horticulturae.

[j_jofnem-2023-0020_ref_009] Elshafie H. S., Camele I., Racioppi R., Scrano L., Iacobellis N. S., Bufo S. A. (2012). *In vitro* antifungal activity of *Burkholderia gladioli* pv. *agaricicola* against some phytopathogenic fungi. International Journal of Molecular Sciences.

[j_jofnem-2023-0020_ref_010] FAOSTAT (2021). Citrus production in the world.

[j_jofnem-2023-0020_ref_011] Ferguson A. R., Testolin R., Huang H., Ferguson A. R. (2016). World economic importance. The kiwifruit genome.

[j_jofnem-2023-0020_ref_012] Gamalero E., Glick B. R., Maheshwari D. (2011). Mechanisms used by plant growth-promoting bacteria. Bacteria in agrobiology: plant nutrient management.

[j_jofnem-2023-0020_ref_013] Garcia C. V., Quek S.-Y., Stevenson R. J., Winz R. A. (2011). Characterization of the bound volatile extract from baby kiwi (*Actinidia arguta*). Journal of Agricultural and Food Chemistry.

[j_jofnem-2023-0020_ref_014] Hao Z., Fayolle L., van Tuinen D., Chatagnier O., Li X., Gianinazzi S., Gianinazzi-Pearson V. (2012). Local and systemic mycorrhiza-induced protection against the ectoparasitic nematode *Xiphinema index* involves priming of defence gene responses in grapevine. Journal of Experimental Botany.

[j_jofnem-2023-0020_ref_015] Hernández-Pacheco C. E., del Carmen Orozco-Mosqueda M., Flores A., Valencia-Cantero E., Santoyo G. (2021). Tissue-specific diversity of bacterial endophytes in Mexican husk tomato plants (*Physalis ixocarpa* Brot. ex Horm.), and screening for their multiple plant growth-promoting activities. Current Research in Microbial Sciences.

[j_jofnem-2023-0020_ref_016] Hu H., Wang C., Li X., Tang Y., Wang Y., Chen S., Yan S. (2018). RNA-Seq identification of candidate defense genes targeted by endophytic *Bacillus cereus*-mediated induced systemic resistance against *Meloidogyne incognita* in tomato. Pest Management Science.

[j_jofnem-2023-0020_ref_017] Izuogu N., Gbenle M., Yakubu I., Abolusoro S. (2015). Reaction of some selected soybean varieties (*Glycine max* (L) Merril) to root-knot nematode infection. Ethiopian Journal of Environmental Studies and Management.

[j_jofnem-2023-0020_ref_018] Lecouls A.-C., Bergougnoux V., Rubio-Cabetas M.-J., Bosselut N., Voisin R., Poessel J.-L., Faurobert M., Bonnet A., Salesses G., Dirlewanger E. (2004). Marker-assisted selection for the wide-spectrum resistance to root-knot nematodes conferred by the Ma gene from Myrobalan plum (*Prunus cerasifera*) in interspecific Prunus material. Molecular Breeding.

[j_jofnem-2023-0020_ref_019] Lesmes-Vesga R. A., Cano L. M., Ritenour M. A., Sarkhosh A., Chaparro J. X., Rossi L. (2022). Rootstocks for commercial peach production in the southeastern United States: current research, challenges, and opportunities. Horticulturae.

[j_jofnem-2023-0020_ref_020] Li Q., Hou Z., Zhou D., Jia M., Lu S., Yu J. (2022). A plant growth-promoting bacteria *Priestia megaterium* JR48 induces plant resistance to the crucifer black rot via a salicylic acid-dependent signaling pathway. Frontiers in Plant Science.

[j_jofnem-2023-0020_ref_021] Li Y., Zhong Y., Huang K., Cheng Z.-M. (2016). Genomewide analysis of NBS-encoding genes in kiwi fruit (*Actinidia chinensis*). Journal of Genetics.

[j_jofnem-2023-0020_ref_022] Liu Y., Yao S., Deng L., Ming J., Zeng K. (2019). Different mechanisms of action of isolated epiphytic yeasts against *Penicillium digitatum* and *Penicillium italicum* on citrus fruit. Postharvest Biology and Technology.

[j_jofnem-2023-0020_ref_023] Lizardo R. C. M., Pinili M. S., Diaz M. G. Q., Cumagun C. J. R. (2022). Screening for resistance in selected tomato varieties against the root-knot nematode *Meloidogyne incognita* in the Philippines using a molecular marker and biochemical analysis. Plants.

[j_jofnem-2023-0020_ref_024] Ma J.-T., Li D.-W., Liu J.-K., He J. (2021). Advances in research on chemical constituents and their biological activities of the genus *Actinidia*. Natural Products and Bioprospecting.

[j_jofnem-2023-0020_ref_025] Maghdouri M., Ghasemnezhad M., Rabiei B., Golmohammadi M., Atak A. (2021). Optimizing seed germination and seedling growth in different kiwifruit genotypes. Horticulturae.

[j_jofnem-2023-0020_ref_026] Maheshwari D. K., Annapurna K. (2017). Endophytes: crop productivity and protection. Sustainable Development and Biodiversity.

[j_jofnem-2023-0020_ref_027] Mahoonaki F. S., Moghadam E. M., Zakiaghl M., Pedram M. (2023). Penetration and development of *Meloidogyne javanica* on four pistachio rootstocks and their defense responses. Journal of Nematology.

[j_jofnem-2023-0020_ref_028] Mohammadi P., Elif T., Recep K., Şenol K. M. (2017). Potential of some bacteria for biological control of postharvest citrus green mould caused by *Penicillium digitatum*. Plant Protection Science.

[j_jofnem-2023-0020_ref_029] Mostafa F. A., Khalil A. E., Nour El-Deen A. H., Ibrahim D. S. (2018). The role of *Bacillus megaterium* and other bio-agents in controlling root-knot nematodes infecting sugar beet under field conditions. Egyptian Journal of Biological Pest Control.

[j_jofnem-2023-0020_ref_030] Moyes A. B., Kueppers L. M., Pett-Ridge J., Carper D. L., Vandehey N., O’Neil J., Frank A. C. (2016). Evidence for foliar endophytic nitrogen fixation in a widely distributed subalpine conifer. New Phytologist.

[j_jofnem-2023-0020_ref_031] Mukhtar T. (2018). Management of root-knot nematode, *Meloidogyne incognita*, in tomato with two *Trichoderma* species. Pakistan Journal of Zoology.

[j_jofnem-2023-0020_ref_032] Mukhtar T., Hussain M. A., Kayani M. Z., Aslam M. N. (2014). Evaluation of resistance to root-knot nematode (*Meloidogyne incognita*) in okra cultivars. Crop Protection.

[j_jofnem-2023-0020_ref_033] Naylor D., DeGraaf S., Purdom E., Coleman-Derr D. (2017). Drought and host selection influence bacterial community dynamics in the grass root microbiome. The ISME journal.

[j_jofnem-2023-0020_ref_034] Nicotra A., Simeone A., De Vito M. (2003). Research on kiwifruit source of genetic resistance to root-knot and lesion nematodes. Proceedings of the fifth international symposium on kiwifruit.

[j_jofnem-2023-0020_ref_035] Pan L., Zhao X., Chen M., Fu Y., Xiang M., Chen J. (2020). Effect of exogenous methyl jasmonate treatment on disease resistance of postharvest kiwifruit. Food Chemistry.

[j_jofnem-2023-0020_ref_036] Rosier A., Medeiros F. H., Bais H. P. (2018). Defining plant growth promoting rhizobacteria molecular and biochemical networks in beneficial plant-microbe interactions. Plant and Soil.

[j_jofnem-2023-0020_ref_037] Rubio-Cabetas M., Lecouls A., Salesses G., Bonnet A., Minot J., Voisin R., Esmenjaud D. (1998). Evidence of a new gene for high resistance to *Meloidogyne* spp. in Myrobalan plum, *Prunus cerasifera*. Plant Breeding.

[j_jofnem-2023-0020_ref_038] Salesses G., Dirlewanger E., Esmenjaud D., Lecouls A., Grzyb Z. S., Zmarlicki K. (1998). Root knot nematode resistance in Myrobalan plum: inheritance and rootstock breeding perspectives using marker-assisted selection. VI international symposium on plum and prune genetics, breeding, pomology.

[j_jofnem-2023-0020_ref_039] Santos M. L. d., Berlitz D. L., Wiest S. L. F., Schünemann R., Knaak N., Fiuza L. M. (2018). Benefits associated with the interaction of endophytic bacteria and plants. Brazilian Archives of Biology and Technology.

[j_jofnem-2023-0020_ref_040] Saucet S. B., Van Ghelder C., Abad P., Duval H., Esmenjaud D. (2016). Resistance to root-knot nematodes *Meloidogyne* spp. in woody plants. New Phytologist.

[j_jofnem-2023-0020_ref_041] Su L., Shen Z., Ruan Y., Tao C., Chao Y., Li R., Shen Q. (2017). Isolation of antagonistic endophytes from banana roots against *Meloidogyne javanica* and their effects on soil nematode community. Frontiers in Microbiology.

[j_jofnem-2023-0020_ref_042] Taechowisan T., Peberdy J. F., Lumyong S. (2003). Isolation of endophytic actinomycetes from selected plants and their antifungal activity. World Journal of Microbiology & Biotechnology.

[j_jofnem-2023-0020_ref_043] Tanha Maafi Z., Mahdavian S. (1997). Species and physiological races of root knot nematodes (*Meloidogyne* spp.) on kiwifruit and the effect of *M. incognita* on kiwifruit seedlings. Applied Entomology and Phytopathology.

[j_jofnem-2023-0020_ref_044] Taylor A. L., Sasser J. N., Taylor A., Sasser J. N. (1978). Biology, identification and control of root-knot nematodes (*Meloidogyne* species). Biology, identification and control of root-knot nematodes (Meloidogyne species).

[j_jofnem-2023-0020_ref_045] Tran T. P. H., Wang S.-L., Nguyen V. B., Tran D. M., Nguyen D. S., Nguyen A. D. (2019). Study of novel endophytic bacteria for biocontrol of black pepper root-knot nematodes in the central highlands of Vietnam. Agronomy.

[j_jofnem-2023-0020_ref_046] Vetrivelkalai P. (2019). Evaluation of endophytic bacterial isolates against root knot nematode, *Meloidogyne incognita* in tomato under glasshouse condition. International Journal of Current Microbiology Applied Science.

[j_jofnem-2023-0020_ref_047] Whitehead A., Hemming J. (1965). A comparison of some quantitative methods of extracting small vermiform nematodes from soil. Annals of Applied Biology.

[j_jofnem-2023-0020_ref_048] Wicaksono W. A., Jones E. E., Casonato S., Monk J., Ridgway H. J. (2018). Biological control of *Pseudomonas syringae* pv. *actinidiae* (Psa), the causal agent of bacterial canker of kiwifruit, using endophytic bacteria recovered from a medicinal plant. Biological Control.

[j_jofnem-2023-0020_ref_049] Xie S., Zang H., Wu H., Uddin Rajer F., Gao X. (2018). Antibacterial effects of volatiles produced by *Bacillus* strain D13 against *Xanthomonas oryzae* pv. *oryzae*. Molecular Plant Pathology.

[j_jofnem-2023-0020_ref_050] Youssef M., Abd Abd-El-Khair H., El-Nagdi W. M. (2017). Management of root knot nematode, *Meloidogyne incognita* infecting sugar beet as affected by certain bacterial and fungal suspensions. Agricultural Engineering International: CIGR Journal.

